# The Rice Semi-Dwarf Mutant *sd37*, Caused by a Mutation in *CYP96B4*, Plays an Important Role in the Fine-Tuning of Plant Growth

**DOI:** 10.1371/journal.pone.0088068

**Published:** 2014-02-03

**Authors:** Jie Zhang, Xiaoqiang Liu, Shuyu Li, Zhukuan Cheng, Chuanyou Li

**Affiliations:** 1 State Key Laboratory of Plant Genomics, National Center for Plant Gene Research, Institute of Genetics and Developmental Biology, Chinese Academy of Sciences, Beijing, China; 2 National Engineering Research Center for Vegetables, Beijing Academy of Agriculture and Forestry Sciences, Key Laboratory of Biology and Genetic Improvement of Horticultural Crops (North China), Beijing, China; University of Nottingham, United Kingdom

## Abstract

Plant cytochrome P450 has diverse roles in developmental processes and in the response to environmental cues. Here, we characterized the rice (*Oryza sativa* L ssp. *indica* cultivar 3037) semi-dwarf mutant *sd37*, in which the gene *CYP96B4* (Cytochrome P450 96B subfamily) was identified and confirmed as the target by map-based cloning and a complementation test. A point mutation in the SRS2 domain of *CYP96B4* resulted in a threonine to lysine substitution in the *sd37* mutant. Examination of the subcellular localization of the protein revealed that SD37 was ER-localized protein. And *SD37* was predominantly expressed in the shoot apical meristem and developing leaf and root maturation zone but not in the root apical meristem. The *sd37* leaves, panicles, and seeds were smaller than those of the wild type. Histological analysis further revealed that a decrease in cell number in the mutant, specifically in the shoots, was the main cause of the dwarf phenotype. Microarray analysis demonstrated that the expression of several cell division-related genes was disturbed in the *sd37* mutant. In addition, mutation or strongly overexpression of *SD37* results in dwarf plants but moderate overexpression increases plant height. These data suggest that *CYP96B4* may be an important regulator of plant growth that affects plant height in rice.

## Introduction

Dwarfism is one of the most valuable agronomic traits in crop breeding because it affects lodging resistance [1], [2], [3] and grain yield [4]. High-yielding, semi-dwarf plant cultivars, produced by traditional crop breeding using both wheat *Reduced-height1* (*Rht1*) and rice *semid-warf1* (*sd1*) genes enabled the “green revolution” to occur [5], [6]. The reduction in plant height enhances lodging resistance, and improves harvest index (grain/grain plus straw) and biomass production in the semi-dwarf cultivars of wheat and rice [6]. Because of their agronomic importance, dwarf mutants have been extensively studied in many plant species. To date, more than 60 recessive dwarf mutants and 10 recessive semi-dwarf mutants have been identified in rice [7]. Most of the dwarfism genes have been cloned and functionally characterized, and many have been directly used in rice breeding programs. These findings have greatly enhanced our understanding of the molecular and genetic regulation of plant height in rice.

In plant, various classes of phytohormones contribute to the regulation of plant height. The importance of phytohormones in regulating plant height is underlined by the dwarf or semi-dwarf phenotypes in various mutants unable to synthesize or perceive a given hormone. Many identified mutants insensitive to or deficient of brassinosteroids (BR), gibberellins (GA), auxins, and cytokinins (CK) all show characteristic dwarfing phenotypes [8], [9], [10], [11]. Among these phytohormones, GA and BR are revealed to be the most important factors in determining plant height [12], [13]. For example, the genes involved in GA metabolism and signaling, such as *D1* [14], *D18* [15], *D35* [16], *SD1* [17], and *ELONGATED UPPERMOST INTERNODE* (*EUI*) [18], all influence the height of rice plants. And *D2* [19], *D11* [20], *BRD1* [21] and *D61* [22] are the genes involved in BR biosynthesis and signaling pathway. The mutations of all these genes result in dwarf or semi-dwarf phenotypes in the mutant plants. Recently, several studies have described GA/BR-independent dwarf mutants and suggested new mechanisms of dwarfism [23], [24], [25]. *SDD1*, which encodes a plant-specific novel protein, controls plant elongation by regulating cell division in rice [23]. Defects in strigolactone biosynthesis, or perception, result in a high-tillering dwarf phenotype, which is involved in stem elongation [24]. Ramamoorthy et al. reported that a *Ds* insertion in *OsCYP96B4* results in a dwarf phenotype in which plants exhibit defects in cell elongation and pollen germination [25]. This mutant has an aberrant lipid profile and is identified as a novel hormone-independent mutant with normal responses to various phytohormones [25]. They also demonstrated that the *OsCYP96B4* dsRNA knockdown could mimic the dwarf phenotype of the mutant and that the over-expression of *OsCYP96B4* reduced plant height in a transcript dosage-dependent manner [25]. Investigation of this mutant may provide novel insight into the mechanisms of the dwarf phenotype, although the details remain to be clarified.

Some of the genes that regulate plant height were found to encode cytochrome P450 monooxygenases, which belong to a notable and large gene family in plant [26]. In rice, 356 cytochrome P450 genes and 99 related pseudogenes have been identify, but the function of most of them are still unknown [26]. These genes were classified into 10 clades, which are designated by CYP71, CYP72, CYP85, CYP86, CYP51, CYP74, CYP97, CYP710, CYP711, and CYP727 [26]. Members of this gene family play an important role in the biosynthesis and perception of plant hormones such as GA and BRs. For example, the ent-kaurene oxidase KO/CYP701 family and the ent-kaurenoic acid oxidase KAO/CYP88A family are required for GA biosynthesis [27], [28]. *CYP714B1* and *CYP714B2* encode gibberellin 13 oxidases in rice [29]. Also in rice, *EUI* encodes CYP714D1, a GA-deactivating enzyme that reduces the biological activity of GA [18]. Biochemical characterization revealed that *CYP724B1, CYP90B2* and *CYP90D2* are encoded by *D11*, *OsDWARF4* and *D2*, respectively. These genes have roles in brassinosteroid metabolism and influence the height of rice plants [19], [20], [30]. The CYP96 family is a younger cytochrome P450 family compared to CYP86, CYP94 and CYP704 family in the CYP86 clade, which consists of seven families namely 86, 94, 96, 704, 730, 731 and 732 [31]. The Arabidopsis CYP96A subfamily is reported to be involved in fatty acid hydroxylation [32], [33]. The CYP96B subfamily is specific to rice [26], though the function of this subfamily has not yet been revealed.

In this study, we identified and characterized a spontaneous rice dwarf mutant named *semi-dwarf 37* (*sd37*). This is another mutant in *CYP96B4* (point-mutation) in the *indica* cultivar 3037. The *sd37* mutant shows a decrease in number of parenchyma cell in the second leaf sheath, especially in internode cell around the shoot apical meristem (SAM). We determined that *SD37* encodes an ER-localized CYP96B4 protein in which the threonine residue at amino acid position 226 in the SRS2 region is important for its function. Interestingly, the moderately elevated expression level of *CYP96B4* (less than two-fold) when governed by its native promoter in transgenic plants promotes plant growth. In contrast, the strong over-expression of *CYP96B4* (more than two-fold) under the maize ubiquitin promoter reduced plant height in a transcript dosage-dependent manner in transgenic rice. Our results suggest that SD37 may be a regulator with a fundamental function in plant growth and provides valuable information concerning the mechanism of dwarfism regulated by *CYP96B4.*

## Results

### Phenotype characterization of the rice semi-dwarf mutant *sd37*

**Figure 1:**
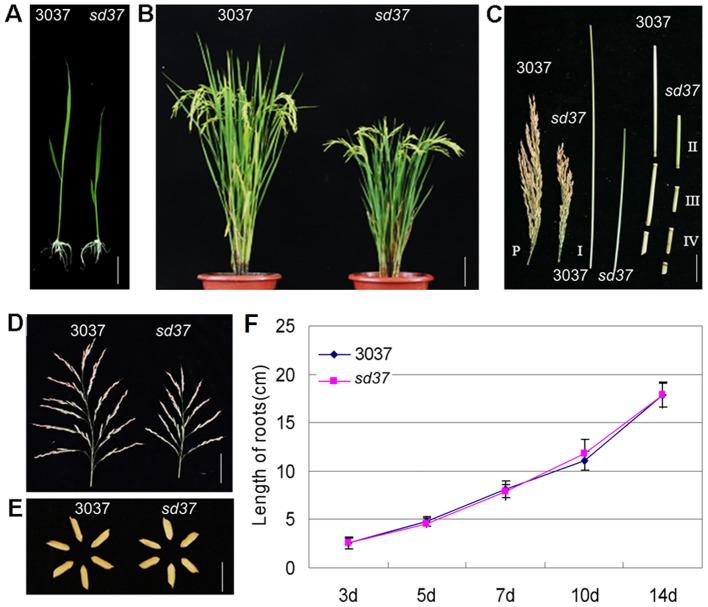
Phenotypic characterization of the *sd37* mutant. (A) Gross morphology of the *sd37* mutant and 3037 (wild type) plants at 7 DAG. Bar  = 1 cm. (B) Heading stage of the *sd37* mutant and 3037 plants. Bar  =  10 cm. (C) Internode lengths of the *sd37* mutant and 3037 plants at the mature stage. P, panicle; I, first internode below panicle; II, second internode below panicle; III, third internode below panicle; and IV, fourth internode below panicle. Bar  =  1 cm. (D) Panicle morphology of the sd*37* mutant and 3037. Bar  =  2 cm. (E) Grain morphology. The *sd37* mutant plants have shorter and broader grains than 3037 plants. Bar  =  5 mm (seeds). (F) Graph showing the root lengths of *sd37* and 3037 plants during the first 14 days of development. Data are averages of 20 plants (± SD).

We identified a spontaneous rice dwarf mutant in the *indica* cultivar 3037 (*Oryza sativa* L ssp. *indica* cv. 3037). This mutant displayed a dwarf phenotype during all stages of development, from seedling to grain filling (Figure 1A, 1B). All internodes of the *sd37* mutant were shorter than those of the wild type (Figure 1C). At the heading stage, the mutant showed a 25–35% reduction in plant height compared to wild-type plants. We thus named this mutant *semi-dwarf 37* (*sd37*). Furthermore, the *sd37* mutant had smaller panicles and shorter rachises than the wild type (Figure 1D). The grains of *sd37* were shorter and wider than those of the wild type (Figure 1E; Table 1). Morphological measurements of the wild type and the *sd37* mutant are shown in Table 1. In contrast, the root length of the mutant was equivalent to that of the wild type in young seedlings (Figure 1F).

**Table 1:** Morphological measurements of the wild-type (3037) and mutant (*sd37*) plants.

Phenotype	3037	*sd37*
Mature plant height (cm)	99.37±4.07	69.82±3.28**
Flag leaf length (cm)	33.80±4.41	18.00±2.51**
Flag leaf width (cm)	1.55±0.08 NS	1.59±0.11
Productive panicle per plant	13.20±5.77**	19.70±5.65
Length of main panicle (cm)	24.90±1.02	16.90±1.15**
Number of grains (per main panicle)	203.80±32.90	96.20±27.50**
Length of seed (mm)	9.63±0.33	8.99±0.46*
Width of seed (mm)	2.81±0.13*	3.28±0.16
Length-width ratio of seed	3.43±0.03	2.74±0.06*
1000-grain weight (g)	25.35±0.30	21.98±0.11**

Data are shown as the mean ± SD (N = 20). Each of the parameters was compared between 3037 and *sd37* using the Student's *t*-test.

### Reduced cell number contributes to the dwarf phenotype of *sd37*

**Figure 2:**
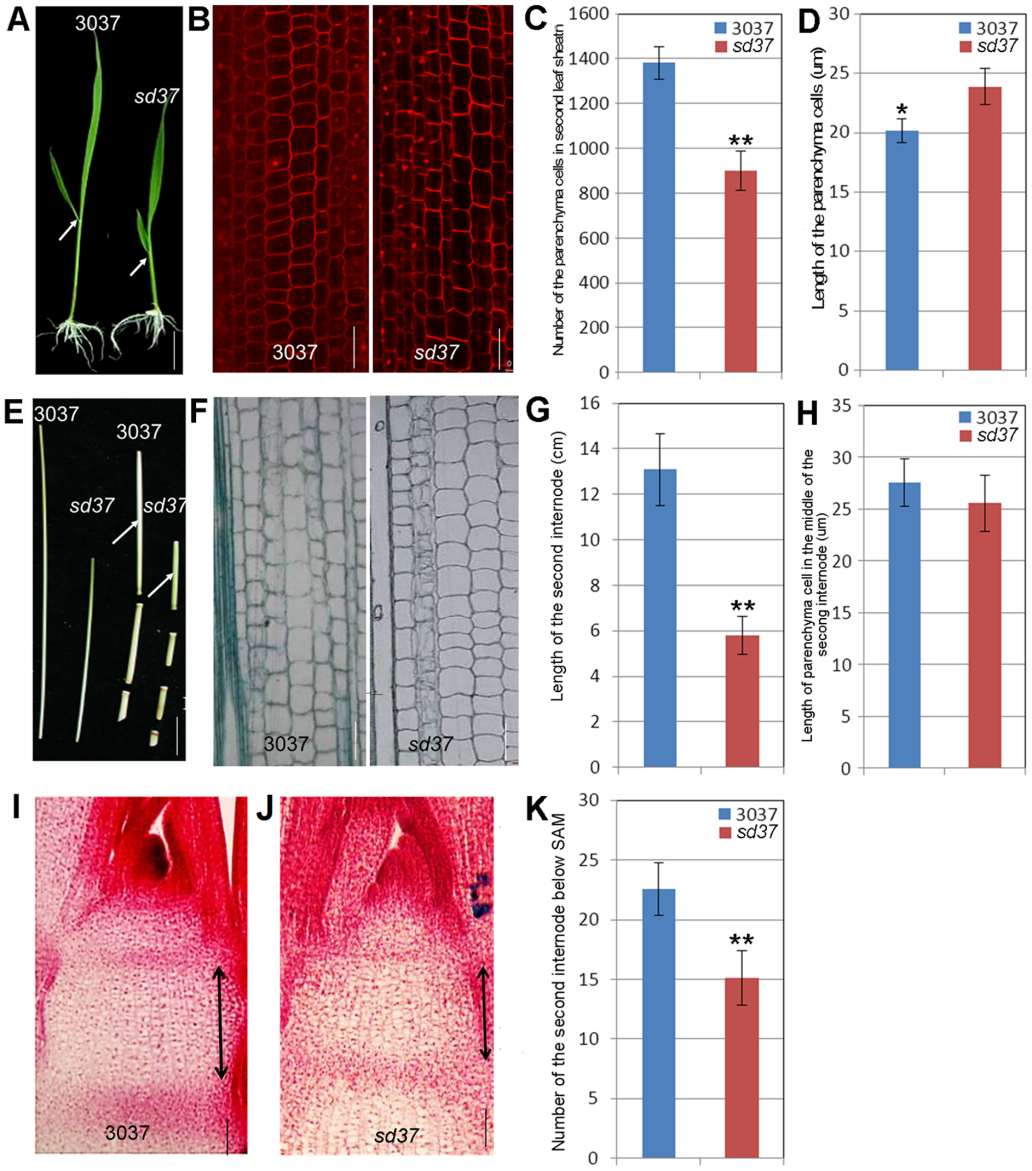
Histological analysis of the aboveground parts of *sd37* and 3037. (A) The seedling phenotypes of *sd37* and 3037 plants at 7 DAG. Arrows indicate the second leaf sheath. Bar  =  1 cm. (B) Parenchyma cells in the second leaf sheaths of *sd37* and 3037 plants. Bars  =  0.05 mm. (C) The number of parenchyma cells in the second leaf sheaths of *sd37* and 3037 plants. Error bars indicate ± SD (N = 20). A significant difference (**, *P*<0.01) was found between the *sd37* and 3037 plants. (D) The length of parenchyma cells in the second leaf sheaths of *sd37* and 3037 plants. Error bars indicate ± SD (N = 20). A significant difference (*, *P*<0.05) was found between the *sd37* and 3037 plants. (E) Longitudinal sections through each stem internode of *sd37* and 3037 plants. Arrows indicate the second internodes below the panicle. Bar  =  1 cm. (F) Longitudinal sections of the middle of the second stem internode of *sd37* and 3037 plants at the heading stage. Bars  =  0.05 mm. (G) The length of the second stem internodes in *sd37* and 3037 plants. Error bars indicate ± SD (N = 20). A significant difference (**, *P*<0.01) was found between the *sd37* and 3037 plants. (H) The length of the parenchyma cells in the second internodes in *sd37* and 3037 plants. Error bars indicate ± SD (N = 20). No significant difference (*P*>0.05) was found between the *sd37* and 3037 plants. (I)–(J) Longitudinal sections through the SAMs of 3037 and *sd37* plants. Arrows indicate the second internode. Bars  =  0.05 mm. (K) Longitudinal cell number in the second internode below the SAM. Error bars indicate ± SD (N = 10). A significant difference (**, *P*<0.01) was found between the *sd37* and 3037 plants.

To explore the underlying cause of the dwarf phenotype in *sd37*, we monitored the number and the morphology of parenchyma cells in the second leaf sheath in both the wild type and the mutant (Figure 2A and 2B). Whereas the *sd37* mutant had 31% fewer cells than the wild type (Figure 2C), the mean length of these cells was greater in the *sd37* mutant (Figure 2D). The cell lengths in the middle of the second internode were compared at the heading stage in both wild-type and *sd37* plants (Figure 2E and 2F). Whereas the second internode was 55.7% shorter in *sd37* than in the wild-type plants (Figure 2G), the length of parenchyma cells in this region were not significantly different (*P*>0.05) (Figure 2H). In addition, we examined the internode cell number around the shoot apical meristem (SAM) (Figure 2I and 2J) and found that the total longitudinal cell number in one internode was 33% lower in the *sd37* mutant than in the wild type (Figure 2K). These histological results suggest that a reduction in cell number is the main cause of the dwarfism phenotype in *sd37*.

### Map-based cloning of *SD37*, which encodes CYP96B4

**Figure 3:**
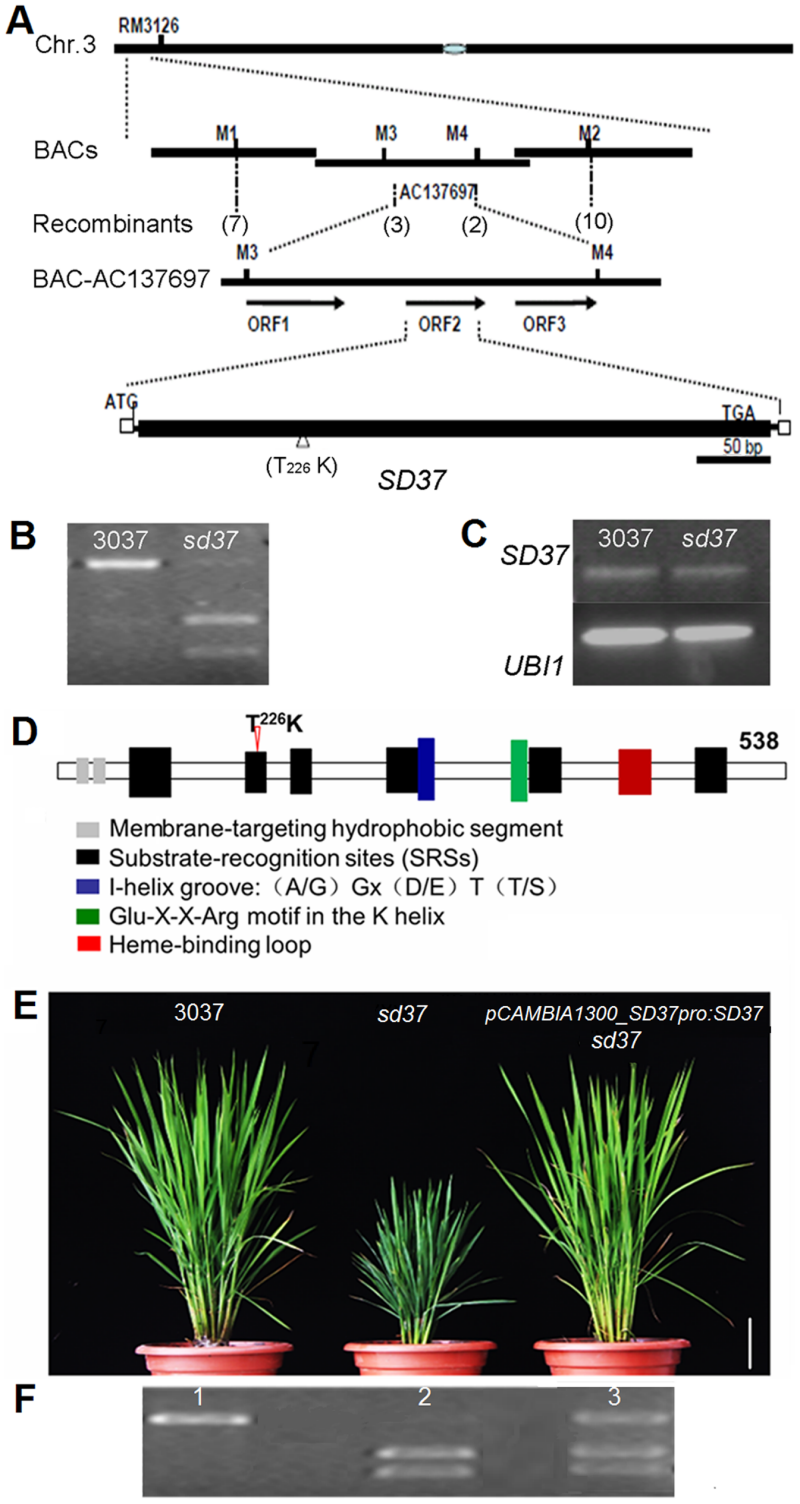
Map-based cloning of SD37. (A) Physical mapping of *SD37*. The numbers in parentheses indicate the number of recombinants. *SD37* was localized to BAC AC13769. The presumed ORFs were predicted using Gramene. White boxes indicate UTRs, and the black box represents the solitary exon. (B) Different sizes of the CAPS markers for 3037 and *sd37* are shown using genomic DNA. PCR products of the *SD37* CDS were amplified using the OE-F and OE-R primers (Table S3) and digested using AlwI. (C) *SD37* expression in leaves from 3037 and the *sd37* mutant were assessed using RT-PCR. Rice *UBQ1* was used as an internal control. (D) Protein structure of SD37. The arrowhead indicates the point mutation in the SRS2 region. (E) Rescue of the *sd37* phenotype with the pCAMBIA1300_SD37pro:*SD37* construct. One representative complementation line (pCAMBIA1300::*SD37*) is shown. Bar  =  10 cm. (F) CAPS marker detection in 3037 (lane 1), *sd37* (lane 2), and a complementation line (lane 3). Samples were analyzed by agarose gel electrophoresis.

To isolate the *SD37* gene, a mapping analysis population was constructed by crossing *sd37* with Nipponbare (*Oryza sativa* L ssp. *japonica*). The mutant phenotype segregated at an approximate 3∶1 ratio in the F2 plants (Chi-squared test), suggesting that this mutation occurs at a single recessive locus. We used 1647 F2 plants to isolate the underlying gene by mapping analysis and established that *SD37* is located in a 30-kb region between the molecular markers M3 and M4 on rice chromosome 3 (Figure 3A). Three open reading frames (ORFs) exist in this region. After sequencing, we identified a single base substitution (C to A) in ORF2. We then designed a CAPS marker to identify this mutation in *sd37* (Figure 3B). The level of *CYP96B4* expression in *sd37* was unchanged compared with that in the wild-type cv. 3037 (Figure 3C).

ORF2 (LOC_Os03g04680) is 1614 bp in length and encodes the putative cytochrome P450 protein CYP96B4, which consists of 538 amino acids (Figure S1) [26]. CYP96B4 contains several conserved domains, including six substrate-recognition sites (SRSs), an I-helix groove, a Glu-X-X-Arg motif, and a heme-binding loop [34]. The mutation discovered in ORF2 resulted in an amino acid substitution (Thr226 to Lys226) in the SAS2 domain, which may impair CYP96B4 function (Figure 3D). The identity of *SD37* was further confirmed by a genetic complementation test. A 4.95-kb genomic DNA fragment containing the entire *CYP96B4* gene and its 2500-bp upstream promoter sequence was cloned into pCAMBIA1300 and introduced into the *sd37* mutant. The *sd37* phenotype was complemented in the resulting transgenic lines (Figure 3E and 3F). Therefore, *LOC_Os03g04680* is the rice *SD37* gene; the described mutation in an exon is responsible for the dwarf phenotype in *sd37.*

### SD37 is localized to the endoplasmic reticulum and predominantly expressed in the shoot meristem

**Figure 4:**
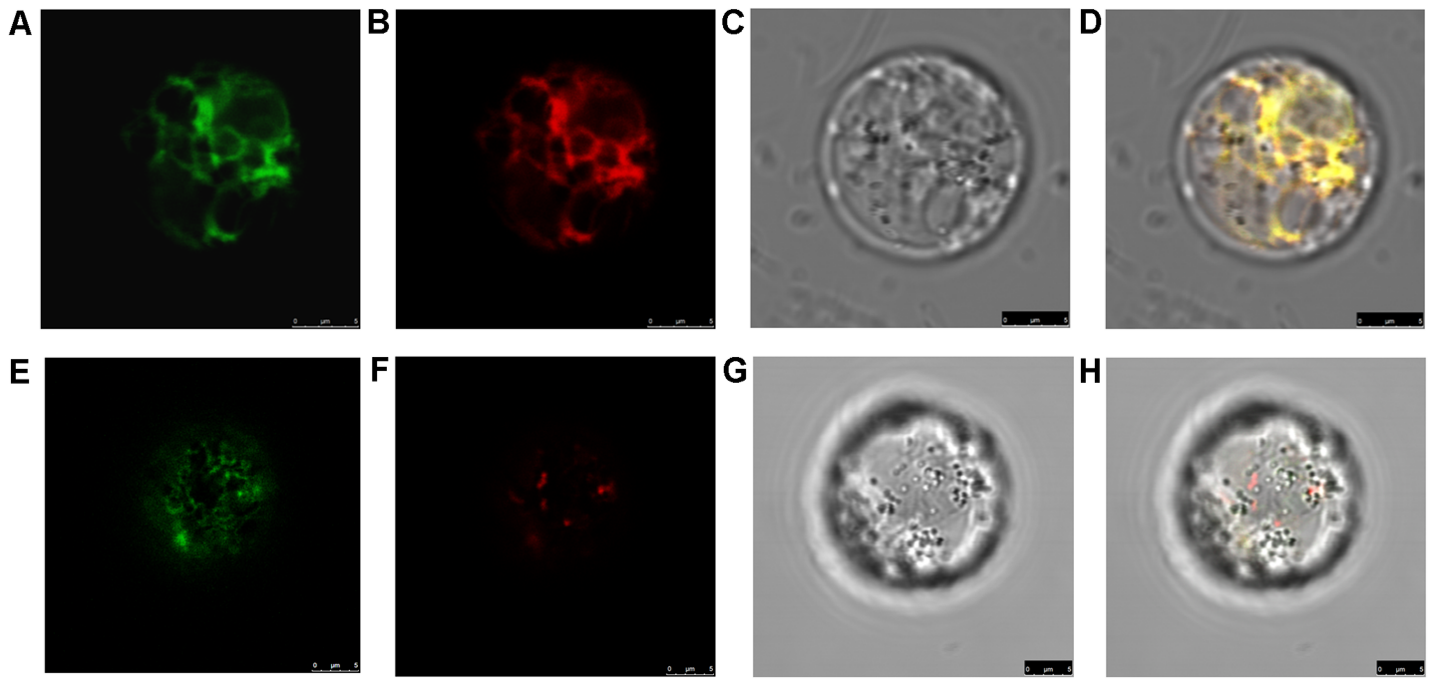
Subcellular localization of pJIT163_hGFP::SD37 using ER-mCherry and Golgi-mCherry in rice protoplast cells. (A) pJIT163_hGFP::SD37. (B) ER-mCherry. (C) Visible light. (D) Merged image. (E) pJIT163_hGFP::SD37. (F) Golgi-mCherry. (G) Visible light. (H) Merged image. Bar  =  5 µm.

Plant P450s are usually anchored to the cytoplasmic surface of the endoplasmic reticulum (ER) and are occasionally associated with plastids [34]. To determine the subcellular localization of SD37, we transiently expressed *SD37* in rice leaf protoplasts. The C-terminus of the SD37 protein was fused with the green fluorescent protein (GFP) under the control of the cauliflower mosaic virus (CaMV) 35S promoter. This plasmid was co-transfected into rice leaf protoplasts with mRFP/mCherry-tagged ER and Golgi markers. As shown in Figure 4, the SD37-GFP fusion protein co-localized with the ER marker (Figure 4 A–D) rather than the Golgi marker (Figure 4 E–H). Thus, SD37 predominantly localizes to the ER.

**Figure 5:**
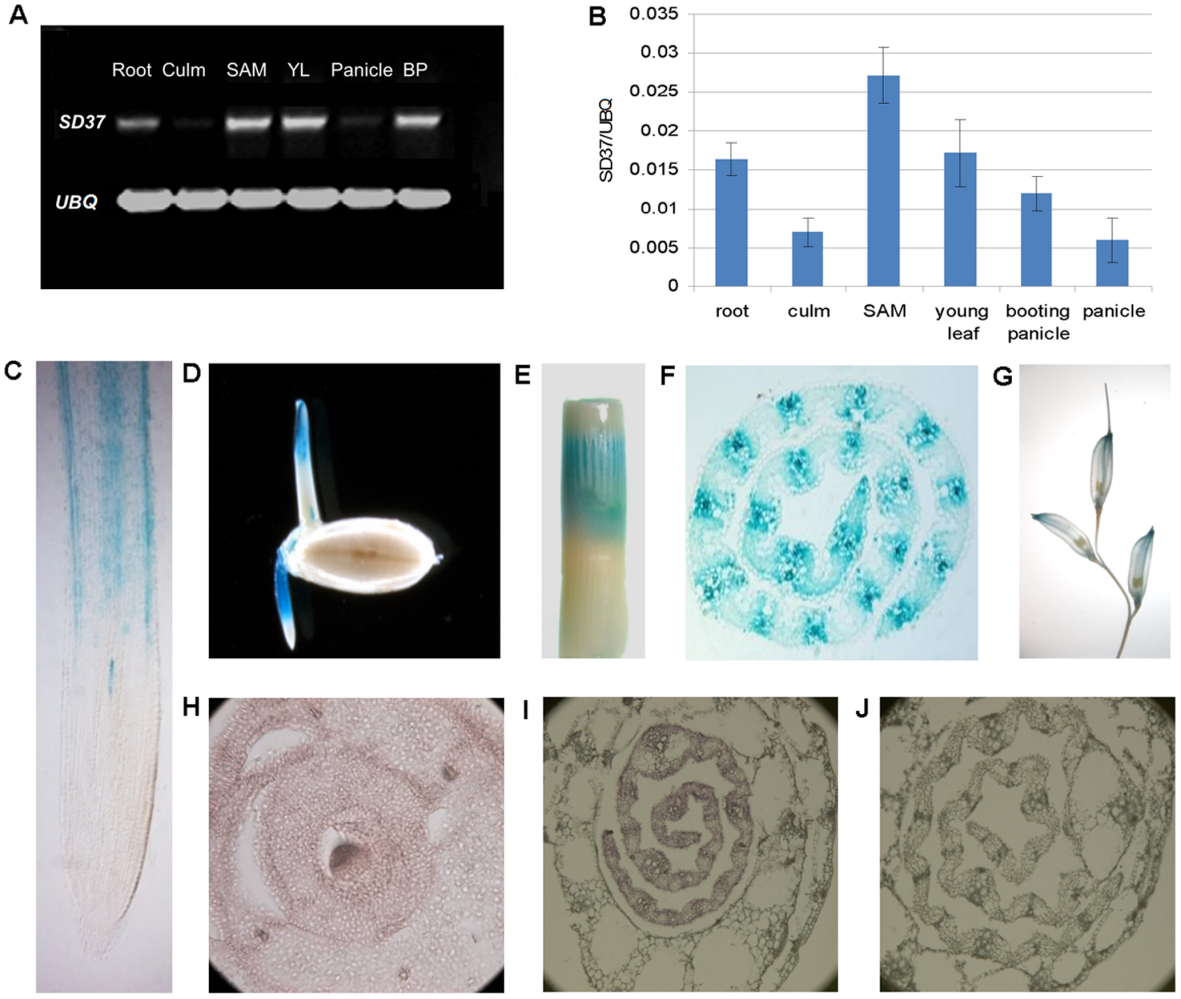
The expression pattern of SD37. (A)–(B) SD37 expression levels were measured by RT-PCR and real-time PCR in various organs, including the root, culm, SAM, young leaf, booting panicle, and panicle. Expression values are the average of 10 samples ± SD. (C)–(G) GUS expression (blue staining) patterns in the p1391Z_SD37pro::GUS transgenic line in different organs. (C) Root cross-section; (D) seeds with coleoptile and radicle; (E) culm; (F) young leaf cross-section; and (G) booting panicle. (H)–(J) *SD37* expression around the shoot apical meristem as revealed by RNA in situ hybridization. (H) Shoot apical meristem; (I) young leaf; and (J) young leaf (negative control) preparation examined with a sense *SD37* probe.

RT-PCR analysis revealed that *SD37* was highly expressed in the SAM (0.028); moderately expressed in young leaves (0.018), root (0.016), and booting panicle (0.013); and expressed at low levels in the mature culms (0.007) and panicles (0.006) (Figure 5A and 5B). The 2.5-kb *SD37* promoter region was amplified and cloned into the pCAMBIA1391Z vector, resulting in a p1391Z_SD37pro::GUS construct. GUS activity was detected in transgenic plants harboring this construct. During the early stages of development, *SD37* was mainly expressed in the root differentiation zone and the coleoptiles (Figure 5D). Low levels of *SD37* expression were detected in the root elongation zone and the root apical meristem (RAM; Figure 5C). During seedling development, strong GUS expression was observed mainly in the intercalary meristem (IM) and in young leaves (Figure 5E, 5F). GUS activity was weak in the culms and panicles (Figure 5E, 5G). To examine *SD37* transcript abundance in the shoot apical meristem, we performed RNA in situ hybridization. Our results showed that *SD37* was abundantly expressed in the SAM and in young leaves (Figure 5H, 5I, 5J).

### The *CYP96B4* mutation influences the expression of certain cell division-related genes

To decipher the function of CYP96B4, a rice whole-genome microarray analysis was performed to monitor the differentially expressed genes in the *sd37* mutant and 3037. In total, 317 differentially expressed genes (1.5-fold cutoff, *P*<0.05) were detected (GEO DataSets, GSE48593). Of these genes, 187 were down-regulated and 130 genes were up-regulated in *sd37* (Table S1).According to Gene ontology (GO), 120 genes were classified in to GO categories. These genes were classified into putative functional categories (biological process, molecular function and cellular component), and the influenced pathways were listed by the enrichment P-values in descending order (Table S2). Through GO term enrichment analysis, we found the nucleobase-containing compound metabolic process, cell cycle and biosynthetic process changed greatly in *sd37* (Table S2). A detailed inspection found the expression level of cell division related genes, such as cell cycle, DNA replication and indole and derivative metabolic process changed obviously. Some cyclin-encoding genes such as *OsCycB1* (*LOC_Os01g59120*), *OsCycD2* (*LOC_Os07g42860*) and the *cdc2* (cyclin-dependent kinase 2) -like genes *LOC_Os06g47310* and *LOC_Os02g06380*) were down-regulated (fold changes of −2.08, −3.85, −3.33, and −2.44, respectively) in the mutant. These genes function as conserved core regulators in the cell cycle [35], [36], [37]. The *OsORC1* (*LOC_Os06g08790*) and *OsMCM3* (*LOC_Os05g39850*) genes, which encode components of prereplication complexes, were down-regulated (fold changes of −1.72 and −3.33, respectively) in *sd37* [38], [39]. The expression level of *OsRPA32* (*LOC_Os02g58220*), which is required for both the initiation and elongation phases of chromosomal DNA replication, was up-regulated 1.84-fold in *sd37* [40]. *EXPB3* (*LOC_Os10g40720*), a member of the β-expansin gene family that plays an important role in cell elongation and cell division, was also up-regulated 1.55-fold in the *sd37* mutant [41]. We also noted that five of the differentially expressed genes were in both the cytoskeleton-related and lipid metabolism pathways. Only two of the differentially expressed genes were in the gibberellin and cytokinin pathways. We further verified the microarray data by real-time quantitative PCR of the genes described above (Table 2).

**Table 2:** Selected functionally classified and differentially expressed genes in the *sd37* mutant compared with the 3037 (wild type) as revealed by microarray analysis.

Gene	Description and functional categories	Fold change (*sd37*/3037)	
		Microarray	qPCR
	**cell division**		
LOC_Os06g08790	origin recognition complex subunit, *OsORC1*	−1.72	−1.37
LOC_Os05g39850	DNA replication licensing factor MCM3	−3.33	−1.94
LOC_Os01g59120	B-type cyclins, OsCycB1	−2.08	−1.59
LOC_Os07g42860	D-type cyclins, OsCycD2	−3.85	−1.26
LOC_Os06g47310	cyclin-dependent cdc2 protein	−3.33	−2.38
LOC_Os02g06380	cyclin-dependent cdc3 protein	−2.44	−2.27
LOC_Os02g58220	replication protein A 32 kDa subunit	1.84	1.50
LOC_Os10g40720	beta-expansin 3	1.55	2.10
	**lipid metabolism**		
LOC_Os03g03370	fatty acid hydroxylase	−1.72	−1.39
LOC_Os08g27040	lipid phosphatase protein	−1.52	−1.85
LOC_Os04g21160	gastric triacylglycerol lipase precursor	8.47	
LOC_Os08g20544	calcium lipid binding protein-like	1.72	2.13
LOC_Os01g22560	glycerol-3-phosphate acyltransferase 1	3.86	
	**cytoskeleton**		
LOC_Os12g42160	kinesin motor domain containing protein	−8.33	−12.54
LOC_Os05g46030	myosin head family protein., OsMyoXIH	1.53	1.20
LOC_Os06g29350	myosin head family protein, OsMyoXIJ	−2.63	−1.55
LOC_Os08g34390	fibroin heavy chain precursor	−14.29	−12.54
LOC_Os10g31720	glycine-rich cell wall structural protein 2 precursor	−14.29	−13.15
	**cytochrome P450**		
LOC_Os03g30420	cytochrome P450 78A11	−8.33	−4.16
LOC_Os12g09790	cytochrome P450 76B1	−4.17	
LOC_Os01g36294	cytochrome P450 71C4	2.05	1.89
LOC_Os07g19160	Cytochrome P450	−4.35	
	**Gibberellin related**		
LOC_Os07g01340	OsGA2ox5	−1.67	−1.50
LOC_Os02g41954	OsGA2ox7	−4.16	−3.20
	**cytokinin related**		
LOC_Os07g30620	cytokinin-O-glucosyltransferase 2,	2.05	2.14
LOC_Os07g30330	cytokinin-O-glucosyltransferase 2	1.95	2.35

### Slight over-expression of *SD37* in transgenic lines promotes plant growth, and RNA interference targeting *SD37* in transgenic lines mimics the mutant phenotype

**Figure 6:**
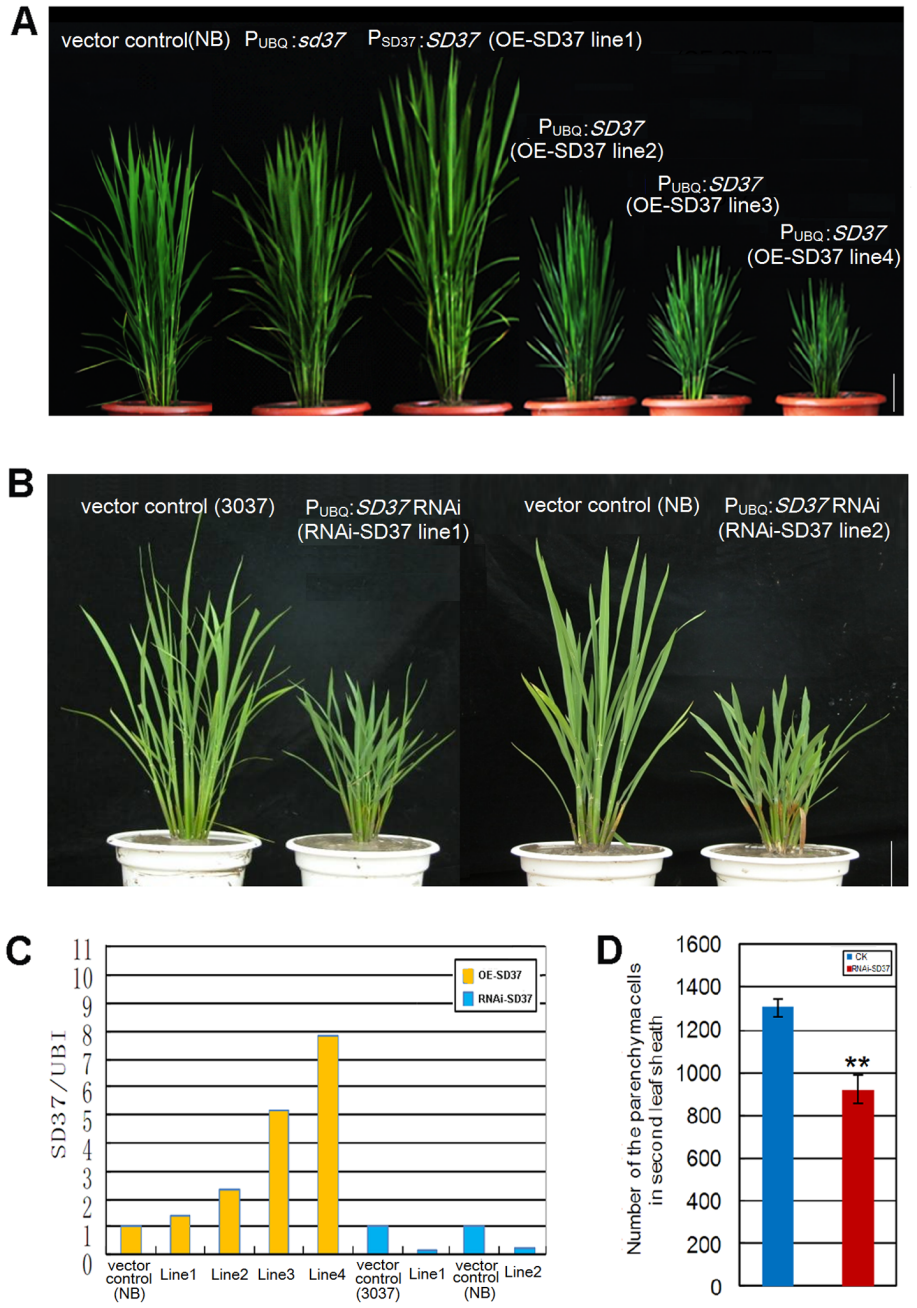
Gross morphology of *SD37* transgenic plants and the relative amount of *SD37* mRNA, as determined by real-time PCR. (A) Gross morphology of the vector-control/Nipponbare; *sd37* over-expressing transgenic line: *P_UBQ_:sd37* in the Nipponbare genetic background; *SD37* over-expressing transgenic line 1:*P_SD37_:SD37* in the Nipponbare background; over-expressing transgenic lines 2–4: *P_UBQ_:SD37* in the Nipponbare background. Bar  =  10 cm. (B) Gross morphology of the *SD37* RNA interference transgenic plants in 3037 and Nipponbare backgrounds at 30 days. Bar  =  10 cm. (C) Relative amount of *SD37* mRNA levels in the transgenic plants in (A) and (B), as determined by real-time PCR. (D) Quantitative measurement of the total second leaf sheath parenchyma cell number in the axial parenchyma cells in the second leaf sheath parenchyma (per leaf) of the RNAi-*SD37* transgenic line and vector control. Error bars indicate ± SD (N = 10). A significant difference (**, *P*<0.01) was found between the RNAi-*SD37* transgenic line and vector control.

We generated a *SD37* promoter-governed *SD37* gene construct and constructs containing *SD37* or its mutant allele *sd37* driven by the maize ubiquitin promoter; these constructs were transferred to Nipponbare to create *SD37* over-expressing transgenic plants (Figure 6A). We monitored the *SD37* transcript levels in independent over-expressing transgenic plants using qRT-PCR (Figure 6C). Our results revealed that the expression level of *SD37* was correlated with a dwarf phenotype in different transgenic lines (Figure 6A and 6C, line 2–4); in these transgenic plants, the expression levels of *SD37* were two-fold greater than in the vector control. However, when the *SD37* expression level was less than two-fold greater than the vector control (1.5-fold), the transgenic plants were taller than the control plants (Figure 6A and 6C, line 1). In total, two independent transgenic lines slightly over expressing *SD37* were observed to be taller than the control plant (Figure S2). Over-expression of the *sd37* (mutant allele) transgenic lines resulted in phenotypes identical to the vector control (Figure 6A). This result suggests that the *sd37* allele had no function and the point mutation in CYP96B4 completely disrupted the catalytic ability of the enzyme. These OE lines with different expression levels suggest that moderate expression of *SD37* promotes plant growth (such as in *OE-SD37* line 1) and high expression of *SD37* (fold change >2) suppresses plant growth.

In addition, we generated transgenic plants expressing the *SD37* RNA interference (RNAi) construct. Similar to the *sd37* mutant plants, the RNAi transgenic plants exhibited a dwarf phenotype (Figure 6B and 6C) with fewer cells found in the second leaf sheath (Figure 6D). Together, these data show that *SD37* plays an essential role in normal plant growth.

### The mutation does not significantly affect pollen viability in the *sd37* mutant

Ramamoorthy et al. [25] observed significantly lower seed viability in the CYP96B4 DS insertion line in a WT background (*Oryza sativa* ssp. Japonica cv. Nipponbare) that was due to defects in pollen viability. We also examined pollen viability and tube growth by iodine/potassium iodide (I_2_/KI) staining and in vitro germination analysis, respectively. As shown in Table 3, 95.4% of 3037 pollen grains were stained; in the *sd37* mutant, 93.7% of pollen grains were stained. An in vitro germination analysis showed that 61.2% of 3037 pollen grains and 58.5% of *sd37* pollen grains germinated. The difference in pollen germination rates between 3037 and *sd37* was not significant (*P*>0.05).

**Table 3:** Pollen viability in the wild-type (3037) and mutant (*sd37*) plants.

Pollen viability	3037	*sd37*
Stained pollen	95.4% NS	93.7%
In vitro germinated pollen	61.2% NS	58.5%

Data are shown as the mean ± SD (N = 100). Each of the parameters was compared between 3037 and *sd37* using the Student's *t*-test.

## Discussion

Previous studies showed that phytohormone GA and BR were the most important regulators in determining plant height. Either the biosynthesis or the perception of GA and BR are revealed to be impaired in many characterized dwarf mutants as introduced earlier. Recently, Ramamoorthy et al. reported a novel semi-dwarf rice mutant, which was a GA/BR independent mutant caused by a copy of Ds insertion into the gene *OsCYP96B4* [25]. And increased expression level of *OsCYP96B4* gene significantly reduced the plant height in a transcript dosage dependent manner [25]. Their data suggested a possible role of OsCYP96B4 gene in fatty acid metabolism. But, the function of OsCYP96B4 gene in regulating plant height is still unknown [25]. Here, we identified a natural semi-dwarf mutant *sd37* and demonstrated that the semi-dwarf phenotype was caused by a point mutation in *OsCYP96B4* gene, which resulted in an amino acid substitution in the CYP96B4 protein. The histological results suggest that a reduction in cell number is the main cause of the dwarfism phenotype in *sd37*. Furthermore, low overexpression of *SD37* promotes plant growth resulting in larger plants, a phenotype not described previously, while transgenic plants in which CYP96B4 is highly overexpressed show a reduced plant height. Ramamoorthy et al. also reported that expression of *OsCYP96B4* transcripts reduced plant height in a dosage-dependent manner in a transgenic plant that contained a *CYP96B4* transgene controlled by its native promoter [25]. Among the three transgenic lines reported in the above study, the E5 line exhibited the lowest transgene expression and a greater than two-fold increase in the *OsCYP96B4* transcript level compared to the vector control [25]; this expression level was similar to what we observed in our line 2. In our present study, an increased plant height was observed in two transgenic lines, such as line 1, compared to the control, and the *CYP96B4* transcript level in these lines was less than two-fold greater than that in the vector control. Our data suggest that moderate expression of *OsCYP96B4* (an increase of less than two-fold) promotes plant growth, but higher expression of *OsCYP96B4* (an increase of more than two-fold) reduces plant height. These data suggest that OsCYP96B4 plays an important role in the fine-tuning of plant growth and that moderate *SD37* expression plays an essential role in the regulation of normal plant growth. Actually, the cytochrome P450 KLUH/CYP78A5 is regarded as a stimulator of plant organ growth in Arabidopsis. Mutation or strong overexpression of *KLUH* results in smaller plants but moderate overexpression increases organ size [42]. Moreover, CYP78A has been proposed to contribute to the biosynthesis of a novel growth-stimulating signal distinct from the classical phytohormones, and some members in CYP78A family were found to catalyze fatty acid hydroxylase reactions [43], [44], [45]. These findings suggest that OsCYP96B4 plays an important role in the fine-tuning of plant growth the same as the AtCYP78A5. In addition, our data provides additional valuable information about the function of OsCYP96B4 to unveil the GA/BR independent pathway which control plant height of rice.

Plant height is determined by cell number and cell size, which depend on cell proliferation and cell expansion, respectively. The *sd37* mutant has fewer parenchyma cells in the second leaf sheath and internode cells around the shoot apical meristem (SAM) compared to the wild type. This result demonstrates that the *sd37* mutant has defects in cell division, which results in its dwarf phenotype. Ramamoorthy et al. [25] reported that the cell elongation defects in the *OsCYP96B4 Ds* insertion mutant line were the major cause of the dwarf phenotype. Their data showed that the average cell length of epidermal cells at the second leaf sheath of the mutant was about 30% less when compared with the wild type. In our research, we did not inspect the cell length of epidermal cells at the second leaf sheath, but we found that the *sd37* mutant is not defective in cell elongation at parenchyma cells in the second leaf sheath and internodes cells around the shoot apical meristem (SAM). In young *sd37* seedlings, the length of parenchyma cells was even greater than that of the wild type. At the heading stage, the decrease in cell elongation in the *sd37* culm internode was not significant compared to that of the wild type. This discrepancy may be due to both the different cell types we analyzed and the different genetic backgrounds and environmental conditions. Ramamoorthy et al. also presented data demonstrating that the heterologous expression of *OsCYP96B4* in *Schizosaccharomyces pombe* led to defects in chromosome segregation that resulted in mis-segregation and wider cells; however, cell length was unaffected [25]. This implied that OsCYP96B4 is able to regulate yeast cell division. Our microarray data also revealed that genes related to cell division were found to be differentially expressed in the *sd37* mutant and the wild type plant. All these data suggested that the cell number is primarily affected by *CYP96B4* mutation, which may have different effect on the regulation of cell growth according to different cell types.

Ramamoorthy et al. observed significantly lower pollen viability in the *CYP96B4 Ds* insertion line in a wild type background (Oryza sativa ssp. Japonica cv. Nipponbare) [25]. We also examined pollen viability and tube growth by iodine/potassium iodide (I2/KI) staining and in vitro germination analysis, respectively. The result showed that pollen germination and iodine/potassium iodide (I2/KI) staining rates in *sd37* was reduced compared with that in 3037, but the difference was not significant (*P*>0.05). This discrepancy may be due to different types of mutants we analyzed. Our mutant allele was a spontaneous rice dwarf mutant in the *indica* cultivar 3037. The *CYP96B4 Ds* insertion mutant used by Ramamoorthy et al. was T-DNA transgenic plant, which usually resulted in reduced fertility [46]. And the different backgrounds and environments may also contribute to this discrepancy. Although *sd37* pollen germinated normally, some over-expression transgenic lines with severe dwarfism (such as OE-SD37 Line 4 in Figure 6) were infertile.

To date, no catalytic or biological function has been assigned to any CYP96B family member. The CYP96 family belongs to the CYP86 clade, which is phylogenetically related to animal and microbial fatty acid hydroxylases. Previous research revealed that the lipid profile in the *OsCYP96B4 Ds* insertion mutant line was different from the wild type [25]. The point mutation of CYP96B4 found in *sd37* may cause a loss of function in enzyme catalysis. Models built for the CYP94 proteins using a hybrid CYP2C9 template indicate that these fatty acid hydroxylases stabilize internal polar/charged groups in their substrates with the polar/charged residues present in the F-helix (SRS2) and the loop between the K-helix and the b1-4 strand (SRS5). In the CYP94B1 model, three charged/polar residues (T226 and Y230 from SRS2 and K373 from SRS5) are important for stabilizing the fatty acid polar groups [47]. It is therefore assumed that the point mutation that led to the substitution of T226 with K226 in the substrate recognition region 2 (SRS2) may interrupt fatty acid metabolism in the *sd37* mutant. We also quantified serials of medium-chain fatty acids in *sd37* mutant and 3037 plant. The result showed that the saturated 16:0 and the polyunsaturated 18:2 levels increased in *sd37* significantly compared with that in 3037 (Figure S3). CYP96B4 did not exhibit catalytic activity toward saturated or unsaturated medium-chain fatty acids in Ramamoorthy's analysis. Using the saturated and unsaturated medium-chain fatty acids (C12, C14, C16, and C18) as substrates, the activity of the recombinant CYP96B4 could not be measured also in vitro in our study (data not shown). Increasing evidence implicates FAs and their derivatives as signaling molecules, modulating normal and disease-related physiologies in plants [48]. The identification of the catalytic substrates of CYP96B4 would be critical to reveal the molecular function of CYP96B4 in regulating plant height in rice.

## Materials and Methods

### Plant materials and growth conditions

The spontaneous mutant *sd37* was isolated from a population of *Oryza sativa* L. ssp. *indica* cv. 3037 at the experimental farm of Yangzhou University, Jiangsu Province, PR China. The *sd37* mutant was crossed with the *japonica* rice variety Nipponbare. Rice plants were cultivated in the experimental field at the Institute of Genetics and Developmental Biology in Beijing under natural growth conditions. The field management adhered to normal agricultural practices. To examine the growth of young rice seedlings, rice seeds were germinated in sterilized water and grown in MS pots in a phytotron chamber with a 16 h light (26°C) and 8 h dark (18°C) photoperiod. For *SD37* expression analysis, booting panicles were collected when they had reached 3 cm in length. The root, SAM, and third leaf sheath were harvested from two-week-old plants.

### Histological observation of cell morphology

Rice samples were fixed in formalin:acetic acid:70% ethanol (1∶1∶18) overnight at room temperature. Each fixed segment was dehydrated then embedded in paraffin wax (Sigma-Aldrich). For the morphogenetic analysis, 8 µm sections were cut using a rotary microtome (Leica). The sections were placed on slides, observed using a microscope, and photographed using a 3CCD color video camera (Leica). The inner layer of the parenchyma cells of the second leaf sheath was stained with propidium iodide (PI) and examined under a laser scanning confocal microscope (Leica TCS SP5). The longitudinal cell number in the layer with the largest cell size was counted for each second sheath per leaf and compared using the Student's *t*-test (N = 20). The cell number was measured in 0.5 mm longitudinal sections from each of the four cell layers in the middle of the second internode bellow the panicle. The cell length was calculated using the average cell number divided by 0.5 mm. The cell length and cell number in these segments (N = 20) were measured and compared using the Student's *t*-test. To measure the cell number in the meristematic internode zone in the SAM, freshly isolated SAMs were embedded in paraffin wax (N = 10) and cut longitudinally. The sections were examined using a microscope and the number of cells in the longitudinal sections of the second internode below the SAM was counted and compared using the Student's *t*-test.

### Map-based cloning and sequencing of *SD37*

To identify the *SD37* gene, the *sd37* mutant was crossed with the japonica cv. Nipponbare. F2 progeny with the mutant phenotype were used to identify the mutation site. Sequence-tagged site markers were designed based on the DNA sequences of *indica* and *japonica* (http://www.ncbi.nlm.nih.gov) and named according to their physical positions. The molecular lesion responsible for the *sd37* phenotype was identified by PCR amplification of the *SD37* genomic region from both 3037 and *sd37* plants; the sequences were compared using DNAMAN. The primer sequences are listed in Table S3. The CAPS markers were generated based on single nucleotide polymorphisms identified in the mutant. PCR products amplified with OE-F and OE-R primers were digested using the restriction enzyme *Alw*I. Digestion products were evaluated by agarose gel electrophoresis.

### Vector construction and Agrobacterium-mediated transformation

For the complementation of the *sd37* mutant, the pCAMBIA1300 plasmid was constructed; this vector contained a 4950-bp genomic DNA fragment consisting of the 2500-bp upstream sequence and the entire *SD37* gene. The plasmid was introduced into the *sd37* mutant.

For the promoter analysis, approximately 2.5 kb of the *SD37* promoter region was amplified. PCR primers were designed with adaptors containing *Bam*HI and *Eco*RI sites. The *SD37* promoter was cloned into the *Bam*Hl and *Eco*RI sites of the promoter fusion vector pCAMBIA139IZ (AF234312) upstream of the GUS gene. For the over-expression analysis, the maize ubiquitin promoter and *SD37* promoter were used to drive the expression of the *SD37* gene. The vector pTCK303 was used to prepare the construct for the RNAi analysis [49]. The agrobacterium-meditated transformation protocol was modified from Hiei [50]. Transgenic plants were selected on medium containing 50 mg/L hygromycin. Hygromycin-resistant plants were transplanted into the soil and the levels of gene expression were assessed.

### RNA microarray analysis and quantitative RT-PCR

The Affymetrix Rice Genome Array contains 51,279 transcripts, including 48,564 japonica transcripts and 1,260 indica transcripts. Two-week-old *sd37* and wild-type seedlings were selected; three biological replicates were generated and evaluated. Total RNA was extracted using the guanidinium isocyanate/acidic phenol method [51]. RNA purification, probe labeling, chip hybridization, probe array scanning, and data pre-processing normalization were performed using the Affymetrix custom services (SBC, Shanghai, China). Analysis was performed using an ANOVA-false discovery rate (ANOVA-FDR) with a significance level of *P*<0.05. Spots with changes in expression were extracted based on a 1.5-fold increase or decrease in expression. Functional classification of the differentially expressed genes was carried out using tools for the GO categories (http://plexdb.org and https://www.affymetrix.com) and revised manually. All of microarray analysis data were submitted to the NCBI Gene Expression Omnibus (GEO) Web Deposit (GSE48593).

After treatment with DNase (Promega), 1 µg of total RNA was used to synthesize the oligo (dT) primed first-strand cDNA using the Invitrogen Super Script III First Strand Synthesis System (Cat. No. 18080-051). All primers used for RT-PCR and qRT-PCR are listed in Table S3. SYBR Green I was added to the reaction system, and reactions were run on a Chromo 4 real-time PCR detection system (Bio-Rad, http://www.bio-rad.com/) with denaturation at 95°C for 10 min, followed by 40 cycles of denaturation at 95°C for 15 s and annealing/extension at 60°C for 1 min. The amplification of ubiquitin gene (*OsUBQ1*) was used as an internal control to normalize the data. These data were analyzed using the Opticon monitor software (Bio-Rad). Three repeats were carried out for each gene.

### GUS staining

GUS staining was performed according to a described previously method [52]. Various tissues or hand-cut sections of p1391z_SD37pro::GUS transgenic plants were incubated overnight at 37°C in a solution containing 50 mM NaHPO_4_ buffer, pH 7.0 with 5 mM K_3_Fe(CN)_6_, 5 mM K_4_Fe(CN)_6_, 0.1% Triton X-100, and 1 mM X-Gluc. The stained sections were then visualized and recorded using a stereomicroscope (Leica). Longitudinal paraffin sections of stained roots and young leaves were observed with a microscope and photographed using a 3CCD color video camera (Leica).

### Subcellular localization

To determine the exact subcellular location of the SD37 protein, *SD37* cDNA was fused in-frame with GFP and ligated into the pJIT163 vector. The fusion proteins were transiently expressed under the control of the CaMV 35S promoter. The expression constructs were co-transfected into rice leaf protoplasts with mRFP/mCherry markers to visualize the endoplasmic reticulum (ER) and Golgi vesicles [53]. The mCherry markers and the pJIT163_hGFP::SD37 construct were transformed into rice leaf protoplasts using the polyethylene glycol method [54]. The transformed protoplasts were examined using a laser scanning confocal microscope (Leica TCS SP5).

### Assessment of pollen viability and tube germination

To analyze pollen development, pollen sampled from *sd37* and wild-type spikelets just before flowering was stained with 1% (w/v) iodine and potassium iodide (I_2_-KI) solution to determine viability. A liquid medium (20% sucrose, 10% PEG, 3 mmol/L Ca(NO_3_)_2_, 10 mg Vitamin B1, and 40 mg/L boric acid) was prepared for rice pollen germination. The optimal incubation temperature was 28°C. The stained pollen grains and the in vitro germination rates were then examined using a Leica microscope (N = 100). Three repeats were carried out and compared using the Student's *t*-test.

### RNA in situ hybridization

RNA in situ hybridization was performed as described [55]. For the *SD37*-specific probe, a 182-bp fragment was amplified from the cDNA of wild-type plants with the primers listed in Table S3. The PCR products were subcloned into the pGEM-T Easy vector (Promega) and used as a template to generate RNA sense probes. Antisense RNA probes were generated in a reaction mixture containing digoxigenin-UTP using T3 or T7 polymerase, depending on the orientation of the inserts. Shoot apices from 3037 and *sd37* plants at different developmental stages were fixed in a formaldehyde solution (4%), dehydrated through an ethanol series, embedded in paraffin (Sigma-Aldrich), and sectioned at 8 µm using a rotary microtome (Leica). Transverse sections were probed with digoxigenin-labeled antisense probes (Roche). The slides were observed using a microscope and photographed using a 3CCD color video camera (Leica).

## Supporting Information

Figure S1 **CDS sequence of** ***SD37*** **. Arrows indicate the point mutation (C to A) in the** ***SD37*** **exon.** A sequence comparison revealed an amino acid substitution of T to K in SD37.
(PPT)

Figure S2 **The phenotype (A) and** ***SD37*** **expression level (B) of over-expressing transgenic plant lines (** ***P_SD37_: SD37*** **in the Nipponbare background).** Bar  = 10 cm.
(TIF)

Figure S3 **Relative level of different fatty acyl chain length lipid from 3037 (wild type) and** ***sd37*** **.** Quantification was made by GC-MS using an internal standard fatty acid C17:0. Error bars indicate ± SD (N = 20). A significant difference (*, *P*<0.05) was found between the *sd37* and 3037 plants.
(TIF)

Table S1 **Up- and down-regulated genes in the** ***sd37*** **mutant, as determined by microarray analysis.** Two-week-old *sd37* and wild-type seedlings were selected. These biological replicates were generated and evaluated, and 317 differentially expressed genes (1.5-fold cutoff, *P*<0.05) were detected.
(XLS)

Table S2 **Gene ontology (GO) enrichment analysis of the up- and down-regulated genes.**
(XLS)

Table S3 **List of primers used for genotyping, probe synthesis, cloning, and expression analysis.**
(XLS)
