# Supplementary material for: The Rice Semi-Dwarf Mutant sd37, Caused by a Mutation in CYP96B4, Plays an Important Role in the Fine-Tuning of Plant Growth
Source: PLoS One. 2014 Feb 3;9(2):e88068. doi: 10.1371/journal.pone.0088068 (PMC3912173; doi:10.1371/journal.pone.0088068)
Supplement: Figure S1 — CDS sequence of SD37 . Arrows indicate the point mutation (C to A) in the SD37 exon. A sequence comparison revealed an amino acid substitution of T to K in SD37. (PPT) [file pone.0088068.s001.ppt]

## Slide 1
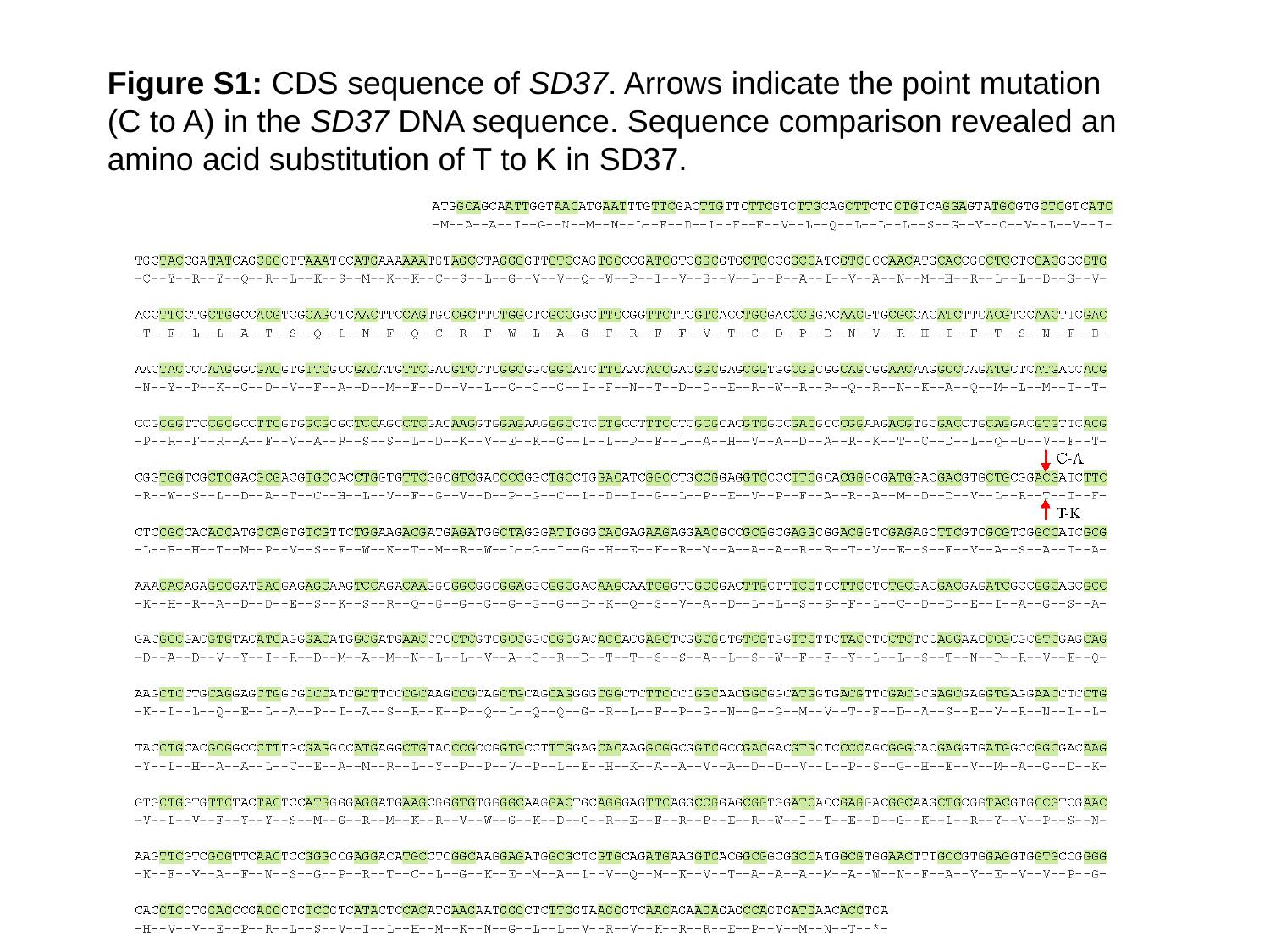

Figure S1: CDS sequence of SD37. Arrows indicate the point mutation (C to A) in the SD37 DNA sequence. Sequence comparison revealed an amino acid substitution of T to K in SD37.
