# Supplementary figures and images for: The Rice Semi-Dwarf Mutant sd37, Caused by a Mutation in CYP96B4, Plays an Important Role in the Fine-Tuning of Plant Growth
Source: PLoS One. 2014 Feb 3;9(2):e88068. doi: 10.1371/journal.pone.0088068 (PMC3912173; doi:10.1371/journal.pone.0088068)

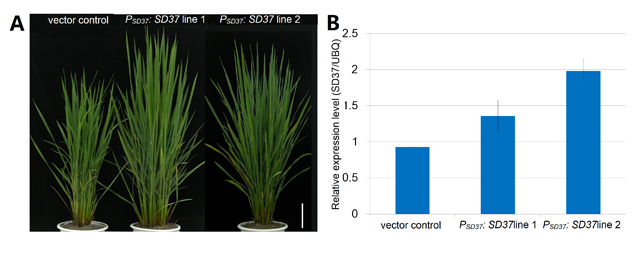

Supplement: Figure S2 — The phenotype (A) and SD37 expression level (B) of over-expressing transgenic plant lines ( PSD37: SD37 in the Nipponbare background). Bar = 10 cm. (TIF) [file pone.0088068.s002.tif]

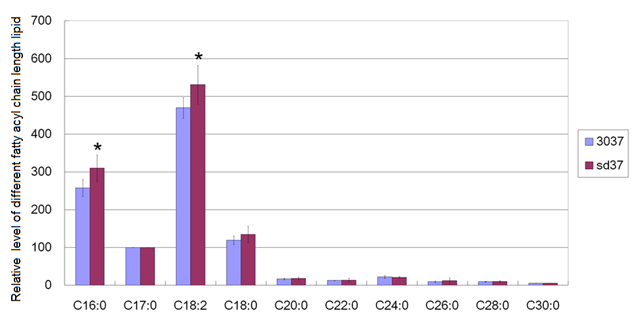

Supplement: Figure S3 — Relative level of different fatty acyl chain length lipid from 3037 (wild type) and sd37 . Quantification was made by GC-MS using an internal standard fatty acid C17:0. Error bars indicate ± SD (N = 20). A significant difference (*, P<0.05) was found between the sd37 and 3037 plants. (TIF) [file pone.0088068.s003.tif]
